# Assessment of prophylactic bone grafting effect on union of open tibial fracture

**DOI:** 10.12669/pjms.291.2722

**Published:** 2013

**Authors:** Mohammad Fakoor, Naser Sarrafan, Naser Naghizadeh-Tabrizi, Morteza Fakoor

**Affiliations:** 1 Mohammad Fakoor, Associate Professor of Orthopedic Surgery, Trauma Research Center, Department of Orthopedic Surgery, Imam Khomeini Hospital, Ahvaz, Iran.; 2 Naser Sarrafan, Associate Professor of Orthopedic Surgery, Trauma Research Center, Department of Orthopedic Surgery, Imam Khomeini Hospital, Ahvaz, Iran.; 3 Naser Naghizadeh-Tabrizi, Orthopedic Surgeon, Trauma Research Center, Department of Orthopedic Surgery, Imam Khomeini Hospital, Ahvaz, Iran.; 4 Morteza Fakoor, Orthopedic Surgeon, Ahvaz Jundishapur University of Medical Sciences, Ahvaz, Iran.

**Keywords:** Open tibial fracture, Autogenus bone graft, Time of union, Infection

## Abstract

***Objective: ***The fracture of the tibial shaft is the most common fracture of long bone in human. Considerable proportion of this fractures are open fractures. Treatment of open fractures is one of the orthopedic problems. In the developing country with economic problem, early mobilization and returning to work may be important for people. We compared result of treatment with addition of autogenus bone graft in two different time periods in two groups.

***Methodology:*** In this study, 144 patients with open tibial fracture were randomly divided in two groups and were treated with autogenus bone graft at two different time intervals, the first group in the end of third week and second group in the end of sixth week. All Patients were followed up periodically in first two month every month and then every two weeks. T-test was used for comparison. SPSS ver. 13.0 (SPSS Inc, Chicago, IL, USA) was used for analysis.

***Results:*** The mean fracture healing time in the first group (with bone graft in 3rd week) was 14.24±4.4 week and in the second group (with bone graft in 6th week) was 16.4±5.4 week and the difference was statistically meaningful. Differences in the rate of delayed union and none union in two groups were statistically insignificant. In addition to time of bone graft, the age, gender, injury mechanism, fixation method, cigarette smoking and drug abuse were studied in two groups. The difference as regards these factors in two groups was statically insignificant.

***Conclusions:*** Achievement of autogenus bone graft in open tibial fracture at the end of third week causes reduction of union time from 16.4 week to 14.4 week without increment of deep infection.

## Introduction

 Tibial fracture is the most common type of long bone fracture with a incidence about 26/100000.^1^ Behrens et al^2^ reported an incidence of open tibial fractures 2/1000 injuries per year in a defined population of industrialized western society; this is 2% of all injury. Classification of open fractures were according to Gustilo classification.^3^ Treatment of open fractures is one of the orthopaedic problems. These fractures are associated with several problems such as soft tissue injury, non union, and osteonecrosis.^4^^-^^6^ Islamic Republic of Iran has the highest rate of mortality in road traffic accidents.^7^ We have no accurate incidence of tibia fracture as the most common long bone fracture in our country. However, it is expected that we have the highest incidence of tibia fracture compared to other countries. We decided to compare autogenus bone grafting in two times to define the better time for this treatment procedure using Gustilo classification for open wound fracture, in view of high tibia fractures and related complication such as non union and infection.^3^^,^^8^^,^^9^

**Table-I:** Age distribution of cases.

*Years*	*Group I*	*Group II*
<=20	17(29.3%)	10(16.1%)
21-30	20(34.5%)	21(33.9%)
31-40	9(15.5%)	7(11.3%)
41-50	5(8.6%)	14(22.6%)
>50	7(12.1%)	10(16.1%)

## Methodology

 In this prospective study, 144 cases with open tibia fracture were initially included. All cases had fracture which were similar to type B and C OTA (Orthopedic Trauma Association). Duration of study was from September 2009 to September 2011. Patients were randomly divided into two groups using random number. Age, sex, and type of injury were matched in two groups. Patients with history of corticosteroid, calcium channel blocker, and local gross infection were excluded from study, because, corticosteriod^10^ and calcium channel blocker^11^ have negative effect on bone healing. By excluding 24 cases with incomplete follow up finally 56 cases in group I and 62 cases in group II were evaluated.

 Endogenous corticocancelous bone grafting was done in the 3rd week in the 1st group and at the 6th week in the 2nd group. Patients were followed in the 1st week, then, monthly for the 1st 2 months and then every two weeks. Anterior-posterior and lateral x-ray was obtained on each visit. Union status was determined according to visibility of the callus in the graph and painless weight bearing. SPSS version 13.0 (SPSS Inc, Chicago, IL, USA) was used for analysis. Independent Sample t-Test, Chi- square, and ANOVA were used for analysis.

 Plate, intramedullary nailing, and external fixator were used for fracture fixation. Plating was done using biologic, locking, and dynamic compression methods. All cases underwent surgery within 24 hours after injury.

## Results

 Out of 58 cases in (group I), 49(84.48%) were male and 9(15.52%) cases were female. In 62 cases (group II), 50(80.64%) were male, and 12(19.36%) were female. There was no significant difference between two groups regarding sex of the patients (P=0.5). Age distribution is shown in Table-I. In the group I and II, 51(87.93%) and 46(74.19%) cases had injury due to road traffic accident. Distribution of fractures and treatment of the two groups is shown in Table II and III.

 Single tibia fracture was present in 47(81.04%) of group I and 52(83.88%) of group II (P>0.05). Mean union time in the first group and 2nd group was 14.24±4.4 wk and 16.4±5.4 wk respectively (p=0.016). Of 58 cases in group I, union was observed in 56(96.6%) of cases before 24 wk. Among 1^st^ group of patients, one case (1.7%) showed delayed union (24-32 wk) and one case showed non union. Of 62 cases in group II, union was observed in 57(91.9%) of cases before < 24 wk. Delayed union was observed in 3(4.8%) of cases. Non union was present in 2(3.2%) of case. There was no significant difference between two groups as regards delayed union.

## Discussion

 In this study, deep infection was not reported. In Blick et al study, deep infection was reported in 20% of cases.^12^ In their study,^12^ 79% of cases had Gustilo type III fracture while in our study, 20% of cases had similar type of fracture. This difference may be the cause of significant difference in deep infection.

**Table-II:** Distribution of fracture types among two groups.

*Type of Fracture*	*Group I*	*Group II*
I	36(62.0)	32(51.6%)
II	15(26%)	13(20.9%)
IIIa	4(6.9%)	13(20.9%)
IIIb	3(5.1%)	4(6.6%)s
Total	58(100%)	62(100%)

In the study by Thakur et al, bone grafting was done for 44 cases with a mean time of 16 weeks after injury.^13^ Infection rate was zero.^13^ The result of two studies was similar as regards infection.

 In our study, mean time of bone union was 14.24±4.4 (group I) and 16.4±5.4 (group II). In Trophet et al. study, in cases with type IIIb fractures, mean time of bone union was 24 wk.^14^ However the results of union times in our study were less than Trophet et al^14^ study, but most of the cases in our study had type I and type II fracture while in Trophet et al^14^ study, fractures were type IIIb. In our study, all cases with rigid fixation were grafted regardless of fracture configuration.

 In Behrens et al study, endogenous bone graft was performed within the 1st four week after injury.^15^ Mean union time was 12 week which is less than our study. In another study on 20 cases with open tibial fracture, mean union time was 28 weeks.^16^ Early bone grafting (<3 months) yielded early bone union than late bone grafting.^17^

 In Fischer et al. study, bone grafting was done before and after 12 wk for two groups of cases respectively.^18^ Mean time of union was 63 wk for the 1st group and 54 wk for the 2nd group.^18^ Mean time of union in our study was less than Fischer et al. study.^18^ This difference may be due to different type of fracture and time of bone grafting in two studies.

**Table-III:** Type of treatment in both groups

*Treatment Method*	*Group I*	*Group II*
DCP Plate	15	15
Intramedullary nailing	28	21
External fixator	8	18
Locking plate	4	5
Biologic plate	3	3

 There is evidence regarding benefit of early prophylactic bone graft for the treatment of tibial open fractures.^12^^,^^16^^,^^17^ Some authors perform bone grafting six months post operatively.^19^ However, there are several controversies regarding this treatment. Further studies are needed in for developing countries with economic problem.

In most of the studies, bone grafting was used for type III fractures. But most of the cases in our study had Gustilo type II. We performed bone grafting earlier than others. This difference may be the cause of different result.

## Conclusions

With endogenous bone grafting in the 3rd week, time of union was significantly less than 6th week of bone grafting. There was no significant increase in deep infection following bone grafting. It is recommended to conduct another study to evaluate using early bone graft to achieve early mobilization and returning to work. 

## Notes

***Limitation of the study:*** Short duration of study was the main limitation.

## Notes

***Acknowledgement:*** This study was supported by Department of Research Affairs of Ahvaz Jundishapur University of Medical Sciences.

## References

[1] Court-Brown CM, McBirnie J (1995). The epidemiology of tibial fractures. J Bone Joint Surg Br.

[2] Behrens F, Searls K (1986). External fixation of the tibia. Basic concepts and prospective evaluation. J Bone Joint Surg Br.

[3] Gustilo RB, Anderson JT (1976). Prevention of infection in the treatment of one thousand and twenty-five open fractures of long bones: retrospective and prospective analyses. J Bone Joint Surg Am.

[4] Audige L, Griffin D, Bhandari M, Kellam J, Ruedi TP (2005). Path analysis of factors for delayed healing and nonunion in 416 operatively treated tibial shaft fractures. Clin Orthop Relat Res.

[5] DeLee JC, Stiehl JB (1981). Open tibia fracture with compartment syndrome. Clin Orthop Relat Res.

[6] Huang CK, Chen WM, Chen TH, Lo WH (1997). Segmental tibial fractures treated with interlocking nails. A retrospective study of 33 cases. Acta Orthop Scand.

[7] Bhalla K, Naghavi M, Shahraz S, Bartels D, Murray CJ (2009). Building national estimates of the burden of road traffic injuries in developing countries from all available data sources: Iran. Inj Prev.

[8] Wiese A, Pape HC (2010). Bone defects caused by high-energy injuries, bone loss, infected nonunions, and nonunions. Orthop Clin North Am.

[9] Gustilo RB, Mendoza RM, Williams DN (1984). Problems in the management of type III (severe) open fractures: a new classification of type III open fractures. J Trauma.

[10] Waters RV, Gamradt SC, Asnis P, Vickery BH, Avnur Z, Hill E (2000). Systemic corticosteroids inhibit bone healing in a rabbit ulnar osteotomy model. Acta Orthopaedica.

[11] Teofilo JM, Brentegani LG, Lamano CarvalhoTL (2001). A histometric study in rats of the effect of the calcium antagonist amlodipine on bone healing after tooth extraction. Archives of Oral Biology.

[12] Blick SS, Brumback RJ, Lakatos R, Poka A, Burgess AR (1989). Early prophylactic bone grafting of high-energy tibial fractures. Clin Orthop Relat Res.

[13] Thakur AJ, Patankar J (1991). Open tibial fractures. Treatment by uniplanar external fixation and early bone grafting. J Bone Joint Surg Br.

[14] Tropet Y, Garbuio P, Obert L, Jeunet L, Elias B (2001). One-stage emergency treatment of open grade IIIB tibial shaft fractures with bone loss. Ann Plast Surg.

[15] Behrens F, Comfort TH, Searls K, Denis F, Young JT (1983). Unilateral external fixation for severe open tibial fractures Preliminary report of a prospective study. Clin Orthop Relat Res.

[16] Kesemenli CC, Kapukaya A, Subasi M, Arslan H, Necmioglu S, Kayikci C (2004). Early prophylactic autogenous bone grafting in type III open tibial fractures. Acta Orthop Belg.

[17] Trabulsy PP, Kerley SM, Hoffman WY (1994). A prospective study of early soft tissue coverage of grade IIIB tibial fractures. J Trauma.

[18] Fischer M, Gustilo R, Varecka T (1991). The timing of flap coverage, bone-grafting, and intramedullary nailing in. J Bone Joint Surg Am.

[19] Cross WW,3rd, Swiontkowski MF (2008). Treatment principles in the management of open fractures. Indian J Orthop.

